# Optimization of Membrane Condenser Process with PTFE Hollow Fiber Membrane

**DOI:** 10.3390/membranes14060141

**Published:** 2024-06-14

**Authors:** Yue Zhou, Susu Long, Zhaohui Wang, Enrico Drioli, Feng Zhang, Zhaoliang Cui

**Affiliations:** 1 Jiangsu National Synergetic Innovation Center for Advanced Materials (SICAM), Nanjing Tech University, Nanjing 210009, China; zy1406640151@163.com; 2 State Key Laboratory of Materials-Oriented Chemical Engineering, College of Chemical Engineering, Nanjing Tech University, Nanjing 210009, China; 17383137991@163.com (S.L.); zhwang@njtech.edu.cn (Z.W.); 3 National Engineering Research Center for Special Separation Membrane, Nanjing Tech University, Nanjing 210009, China; 4 Research Institute on Membrane Technology, ITM-CNR, Via Pietro Bucci 17/C, 87036 Rende, Italy; e.drioli@itm.cnr.it

**Keywords:** membrane condenser, process parameters, water recovery

## Abstract

A membrane condenser (MC) is a novel membrane separation technology that utilizes the hydrophobic nature of porous membranes to capture water vapor from humid gas. Factors such as temperature, pressure, flow rate, and gas composition entering the membrane condenser play a crucial role in water recovery efficiency. This study utilized hydrophobic polytetrafluoroethylene (PTFE) hollow fiber membranes to create multiple identical membrane modules. This research investigated the impact of temperature, flow rate, pressure on the intake side, gas flow on the cooling side, membrane area, and other variables on the performance of the membrane condenser process. This study compared water extraction efficiency under different conditions, focusing on feed flow temperature and sweeping flow. Results showed that at a temperature of 60 °C, the water recovery rate was 24.7%, while a sweep gas flow rate of 4 L/min resulted in a recovery rate of 22.7%. The efficiency of the membrane condenser decreased with higher feed flow rates but increased with larger membrane areas. A proportional relationship between inlet flow and membrane area was observed, suggesting an optimal range of 0.51–0.67 cm/s for both parameters. These findings offer valuable insights for the practical implementation of hydrophobic membrane-based membrane condenser technology.

## 1. Introduction

With the expansion of the global population and the increased utilization of natural resources, the world is currently grappling with a growing number of challenges related to human health and the environment [1]. The discharge of high-humidity flue gas not only results in significant water resource depletion [2] but also leads to the formation of aerosols through the reaction of sulfur and nitrogen oxides with water vapor, dust, and other particles, causing issues such as white plume [3], gray haze [4,5,6], gypsum rain [7], and low-temperature corrosion [8]. These problems have detrimental effects on industrial production and the surrounding environment. Emission Standards for Air Pollutants from Coal-fired Power Plants (DB 31/963-2016) introduced by Shanghai in 2016 explicitly mandates that coal-fired power boilers must implement measures such as smoke temperature control to eliminate phenomena like gypsum rain and colored smoke plume. This regulation underscores the importance of recycling water in flue gas not only for industrial water self-recycling but also for mitigating the environmental impact of flue gas emissions in industrial settings.

**Figure 1:**
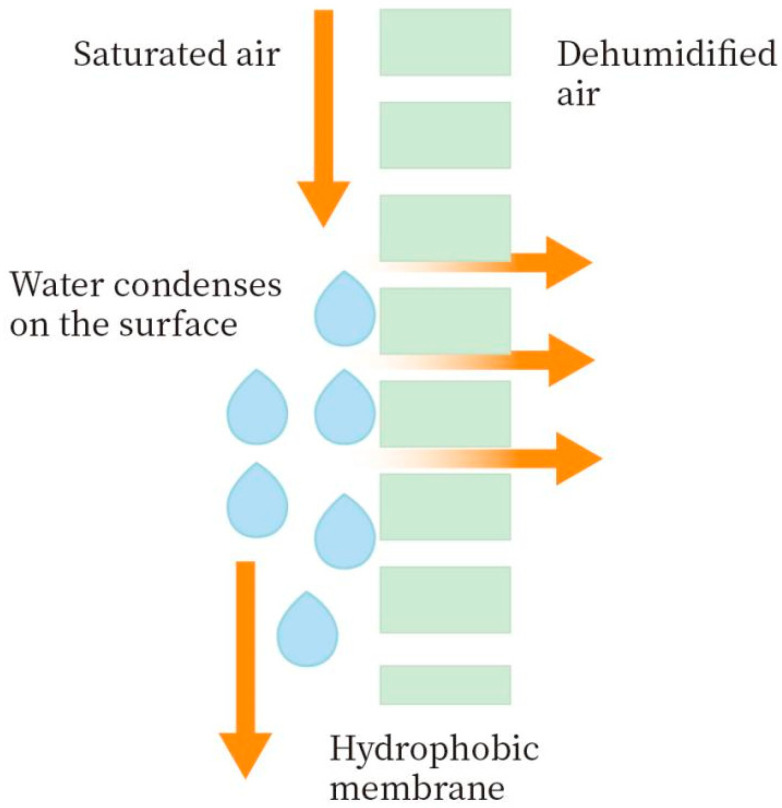
Schematic diagram of membrane condenser.

Membrane technology is an industrial process widely used in seawater desalination [9] and wastewater treatment [10]. With the development of new membrane materials and technology, there has been an increase in studies on using membrane separation technology to recover water vapor from high-humidity gases [11]. Compared with traditional processes, membrane separation technology has the characteristics of continuous operation, no regeneration, no secondary pollution, easy operation, and small footprint, and it is considered an effective alternative technology [12]. The concept of membrane condensers using hydrophobic membranes for water vapor recovery in industrial gases was first proposed by Prof. Drioli’s group in 2013, as shown in Figure 1 [13].

High-humidity flue gas reaches a supersaturated state and contacts the lower-temperature hydrophobic membrane. The roughness of the hydrophobic membrane causes the supersaturated water vapor to condense heterogeneously. Due to the hydrophobic nature of the membrane, the condensed water is trapped on the feed side, while non-condensing gas and non-condensing water vapor permeate through the membrane pore and enter the next side [14]. Brennan et al. [15] conducted a study to investigate how the molecular composition of rendered condensate wastewater changes chemically to make it suitable for treatment using hydrophobic membranes and to create a viable product. The primary goal was to test the effectiveness of ammonia stripping technology using two types of hydrophobic membrane materials, polypropylene (PP) and polytetrafluoroethylene (PTFE), at a pilot scale. The results indicate that PTFE membranes exhibit promising characteristics, boasting a longer lifespan compared to PP membranes and achieving the removal of up to 64% of NH_3_ molecules from condensate waste. This study marks the inaugural use of hydrophobic membrane contactors for condensate wastewater treatment. Brunetti et al. [16] used the PVDF hollow fiber membrane to extract water from simulated flue gas using the MC process, maintaining a consistently high water recovery rate over a 150-day operational period. At Δ*T* of 8 °C, more than 25% water recovery is achieved, and the plant is self-sufficient in cooling circulating water. At Δ*T* of 15 °C, the PVDF membrane can recover more than 60% water vapor from the flue gas. Drioli et al. [17] compared the prepared ECTFE hydrophobic membrane with the commercial PVDF hollow fiber membrane in terms of contact angle, mechanical properties, pore size, and porosity and tested the performance of the two membranes in the membrane condenser under different feed temperatures and feed flow rates. The results showed that the water recovery rate of the two membrane materials was similar.

In 2013, Macedonio et al. [18] investigated the potential of membrane condensing technology in recovering evaporated water in flue gas, which they referred to as MC. The simulation’s effectiveness and applicability were confirmed through experimental analysis, and it was pointed out that the same simulation method is suitable for various high-humidity flue gases. Then, the experiment and simulation were integrated to conduct a more comprehensive analysis. Several main influencing parameters were identified, including gas flow rate, temperature difference, membrane area, and gas flow/membrane area ratio, which were systematically tested and simulated. The experimental data confirm the validity of the simulation study and identify the appropriate membrane condensation based on the simulation operating conditions [19]. In a study by Macedonio et al. [20], a small amount of NH_3_ was introduced into the high-humidity feed gas to mimic the cooling tower plume. Their study investigated the impact of technical parameters such as temperature difference Δ*T* between the feed plume and the membrane element, relative humidity (RH) of the plume, and the ratio of feed flow to membrane area (Q/A) on water recovery performance and NH_3_ concentration in the condensing water. The increase in NH_3_ content, Δ*T*, and relative humidity in the plume will lead to an increase in NH_3_ content in the condensate water on the stranded side. When the plume contained 100 ppm of NH_3_, the plume temperature was 30 °C, and Δ*T* was 6.4 °C; the condensed water collected on the receiving side contained approximately 25 ppm of NH_3_, confirming the possibility of the MC process in recovering chemical pollutants from the waste stream with low energy consumption. 

PTFE hollow fiber membrane exhibits outstanding temperature resistance, strong acid and alkali resistance, aging resistance, pollution resistance, and other properties. The material can be effectively chemically cleaned and solve the problem of membrane fouling [21,22]. PTFE can be a stable operation under poor water quality conditions. In addition, the PTFE hollow fiber membrane has strong mechanical properties and fatigue resistance, reducing the likelihood of fracture and extending the membrane’s useful lifespan during large flow backwashing. Therefore, this study utilized the PTFE hollow fiber membrane for membrane condensation testing.

In practical applications, the temperature, pressure, flow rate, and other factors of gas entering the membrane condenser will also influence the water recovery efficiency of the membrane condenser to varying degrees [23,24]. This study explores various process parameters to determine the optimal conditions for the membrane condensation process, offering theoretical backing for the real-world implementation of hydrophobic membrane-based membrane condensation technology.

## 2. Experiment

### 2.1. Materials

Commercial PTFE hollow fiber membranes were purchased from Zhejiang Dongda Environmental Engineering Co., Ltd. (Zhuji, China) Ethanol (>99.7%) was purchased from Shanghai Lingfeng Chemical Reagent Co., Ltd. (Shanghai, China). Pure water was purchased from Hangzhou Wahaha Group Co., Ltd. (Zhejiang, China). Polyurethane glue was purchased from Shanghai Sanyou Resin Co., Ltd. (Shanghai, China). All chemicals were used without further purification.

In industrial applications, factors such as safety, equipment cost, equipment area, and operating parameters must be considered. Therefore, for industrial scale-up, hollow fiber membrane modules were selected for this experiment. They are characterized by large packing density, easy installation, and small footprint for the entire membrane condenser device. PTFE membrane was selected to conduct membrane condenser experiments.

### 2.2. Membrane Condenser Experiments

**Figure 2:**
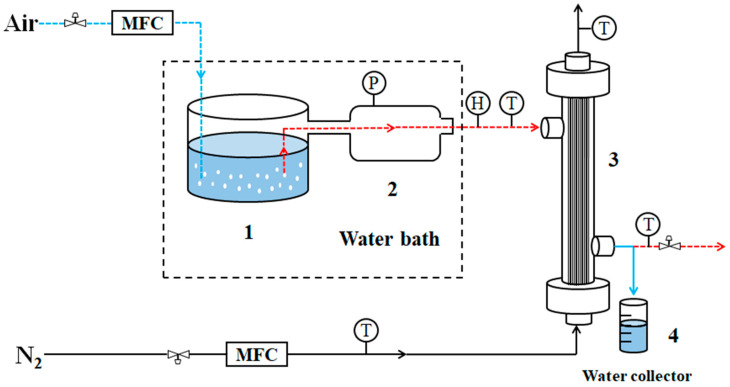
Membrane condenser experimental equipment (1-humidifier, 2-buffer tank, 3-membrane module, 4-beaker, P-pressure gauge, H-hygrometer, T-thermometer, MFC-mass flow controller. Dashed wireframe is heated in a water bath).

Hollow fiber membrane modules offer a large filling area and high heat transfer efficiency, making it the core component of the membrane condenser device shown in Figure 2. Dry air driven by an air compressor is directed into a custom-made humidifier and mixed in a buffer tank to create a high-humidity simulated flue gas. This gas is then heated in a water bath and passed into the feed side of the assembly. Upon contact with the hydrophobic membrane surface, the water vapor in the high-humidity gas condenses into liquid water due to the higher temperature of the gas. Some of the non-condensable gas enters the penetration side through the membrane pore, and the other part of the non-condensable gas is discharged from the inlet side of the assembly. On the permeable side of the membrane assembly, N_2_ cylinder gas is used as the purge gas in the membrane condenser to cool and carry the gas through the feed side. The gas flow forms on both sides are cross-flow, which further improves the heat transfer efficiency of the membrane condenser. 

The experiment replicates high-humidity gas conditions through air humidification, maintaining a relative humidity of 95%. When the relative humidity is low, the humidity of the feed gas can be increased by a steam generator. In order to simulate the temperature of the high-humidity gas on the feed side, humidified air is heated to 45–60 °C using a water bath. Thermal insulation foam is wrapped around the inlet and outlet pipes of the membrane module to prevent water vapor condensation and any impact on test results.

### 2.3. Experimental Process of Membrane Condenser

The self-made membrane condenser experiment system is shown in Figure 2. The gas used primarily consisted of sweeping gas (N_2_), air from an air compressor, and water vapor from a steam generator. Prior to conducting the membrane condenser experiment, the membrane underwent a dehydration process in a vacuum-drying oven. Following the assembly of the experimental apparatus, the water temperature in the constant temperature tank was adjusted to a specific level. Once the water temperature stabilized, the air compressor was activated to allow air to pass through the humidifier for humidification. The air and water vapor were thoroughly mixed in the buffer tank. The humidified gas was then continuously discharged for 1 h to ensure that the temperature and humidity of the gas were consistent upon entering the membrane module.

Before the module is connected, wrap a layer of insulation material outside the module and put it in an oven to keep it warm for 3 h. Adjust the oven temperature to match the intake air temperature to prevent water vapor condensation in the high-humidity gas within the membrane module. Once the temperatures inside and outside the module stabilize, promptly remove it from the oven and connect it to the membrane condenser experiment device. When the inlet and outlet temperatures on the shell side of the membrane module are equal, connect a sweeping gas to the permeate side of the membrane module. After the system is stable and runs continuously for 3 h, measure the mass of condensed water on the interception side. The condensation performance of the membrane is primarily evaluated based on the water recovery rate and the condensate transport flux on the interception side. The water recovery rate can be calculated using the following formula:

(1) $$R=\frac{∆m}{M}$$

where *R* is the water recovery rate, %. ∆*m* is the mass of liquid water on the interception side, kg. *M* is the mass of water vapor contained in the flow rate on the feed side during the same period of operation, kg.

(2) $$J=\frac{∆m}{A\times ∆t}$$

where *J* is the condensate transport flux, kg/(m^2^·h). ∆*m* is the mass of liquid water on the interception side, kg. ∆*t* is the operating time, h. *A* is the effective area of the membrane, m^2^.

In this study, a hydrophobic hollow fiber membrane was made into multiple identical membrane modules, and the influence of temperature, flow rate, pressure on the feed side, gas flow rate on the cooling side, membrane area, and other factors on the performance of the membrane condenser process based on the hydrophobic membrane were investigated. The membrane module is filled with 3 membrane filaments, and the membrane length is 20 cm. To investigate the impact of membrane area, the membrane modules were filled with 1, 2, 3, and 4 membrane filaments, respectively.

### 2.4. Structure and Characterization of Porous Hydrophobic Membranes

#### 2.4.1. Measurement of Membrane Porosity

The porosity of the membrane was characterized by the weighing method. Select a membrane of a certain size, measure the quality of the membrane before it is soaked in kerosene, and then soak the membrane in kerosene for 24 h before taking it out, wipe the residual kerosene on the surface of the membrane with a non-woven cloth or test paper, and then quickly measure the quality of the membrane sample after soaking the kerosene. The porosity is calculated as follows:

(3) $$\varepsilon =\frac{\frac{m_{1}-m_{0}}{\rho_{k}}}{\frac{m_{1}-m_{0}}{\rho_{k}}+\frac{m_{0}}{\rho_{p}}}$$

where *ε* is the porosity of the membrane, %. *m*_1_ is the mass of the wet membrane after wetting, g. *m*_0_ is the mass of dry membrane, g. *ρ*_p_ is the density of PTFE; the value is 2.15 g/cm^3^; *ρ*_k_ is the density of the wetting medium kerosene; the value is 0.82 g/cm^3^. Different membranes prepared under the same conditions were measured five times, and the final measured values were averaged.

#### 2.4.2. Measurement of Membrane Pore Size Distribution

The average pore diameters of the membrane used for condensation were measured using a gas–liquid removal aperture distributor. Initially, the hydrophobic porous membrane was immersed in the wetting agent (GQ-16) for over 1 h to ensure complete wetting. Subsequently, the test membrane sample was extracted, appropriately sized, and prepared for testing. Operating parameters were input into the computer connected to the instrument. Each sample was tested 5 times under identical conditions, recording the maximum and average aperture values, with the average being considered as the test result. 

#### 2.4.3. Water Contact Angle Measurement

The water contact angle of the hydrophobic membrane used in the membrane condenser is measured using a water contact Angle measuring instrument. To conduct the test, the membrane sample is first vacuum dried; then, a suitable size of the membrane is gently pasted onto double-sided tape, and any surface dust is wiped off with a non-woven cloth. The sample was placed on the test bench; 1.0 μL deionized water was dropped on the surface of the flat membrane at room temperature, and the contact angle measuring instrument was used for determination. Take five different positions of each sample for the water contact angle test and take the average value.

PTFE membrane has excellent chemical properties; the basic characteristics are as Table 1. 

## 3. Results and Discussion

### 3.1. Effect of Feed Gas Flux

**Figure 3:**
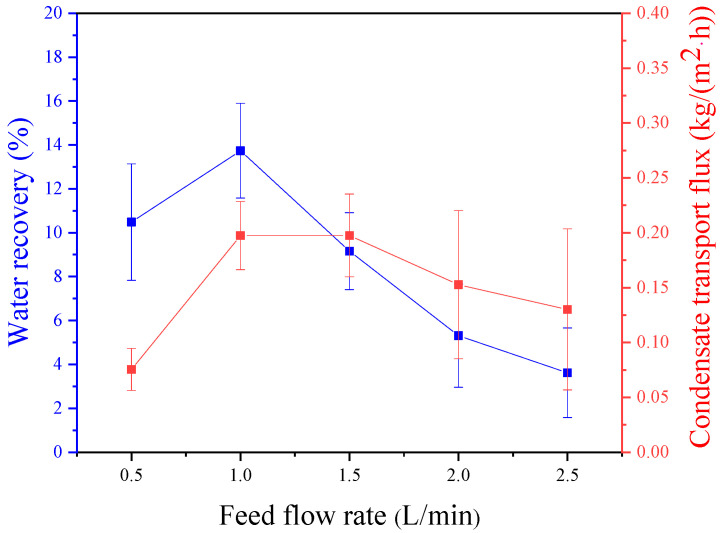
Effect of feed flow rate on water recovery and condensate transport flux across membrane condenser.

The experimental conditions included a relative humidity of 95%, a feed temperature of 50 °C, a pressure difference is 10 kPa, a sweeping gas flux of 2 L/min, a sweeping gas temperature of 26 °C, and a membrane area of 32.78 cm^2^. The impact of feed gas flux on the water recovery rate was examined. As shown in Figure 3, the water recovery rate initially rises and then declines with an increase in feed gas flux. The highest water recovery rate (13.7%) was observed at an intake air flow rate of 1 L/min. The reduction in residence time of the gas in the membrane condenser due to an increase in feed gas flux results in a significant number of water molecules passing through the membrane condenser without condensing. Moreover, with constant sweeping gas flow rate and temperature, the heat load remains constant. As the feed gas flux rises, the number of condensed water molecules per unit time increases, causing a substantial amount of water vapor to be unable to release heat to condense into water, ultimately leading to a decrease in water recovery. When the feed gas flux is 0.5 L/min, water droplets struggle to escape from the membrane wire due to the low feed gas flux, leading to the formation of large droplets on the membrane surface. This reduces the contact area between the gas and the membrane, which is akin to increasing thermal resistance, resulting in a smaller heat transfer coefficient and decreased water recovery. Figure 3 shows the effect of feed gas flux on condensation flow, showing a pattern of initial increase followed by a decrease. This behavior is attributed to the rise in water molecules due to the feed gas flux, causing an increase in the condensation rate of water molecules per unit time. At a flow rate of 0.5 L/min, the minimum flow rate is 0.075 kg·m^−2^·h^−1^. There is minimal variance in the quantity of condensed water across other feed gas flows, indicating that water droplets adhering to the membrane surface significantly affect the membrane condenser performance. Therefore, it is necessary to enhance the feed gas flux to prevent an excessive accumulation of water droplets on the membrane surface. With a feed gas flux of 1.5 L/min, the maximum condensate transport flux is 0.198 kg·m^−2^·h^−1^, only slightly different from the condensate transport flux of 1 L/min (0.197 kg·m^−2^·h^−1^). The only difference is the water recovery rate, making a feed gas flux of 1 L/min optimal for the membrane condensation efficiency.

### 3.2. Effect of Membrane Area

**Figure 4:**
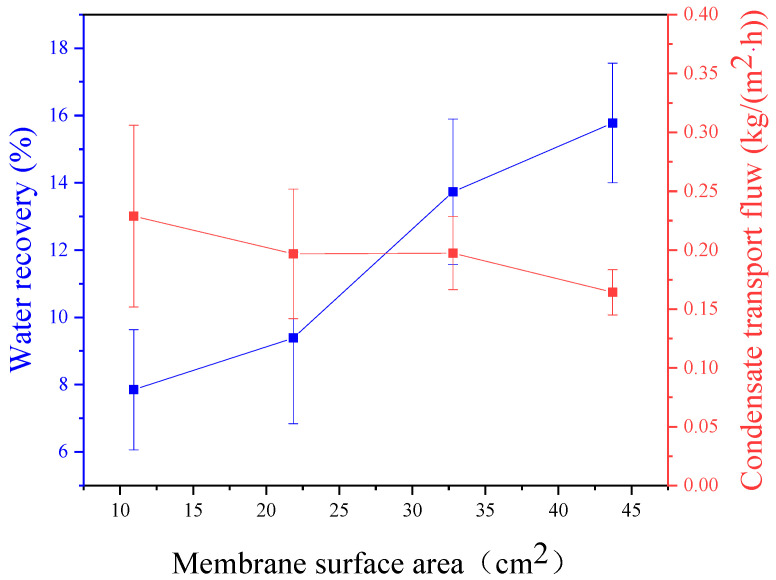
Effect of membrane surface area on water recovery and condensate transport flux across membrane condenser.

This experiment was conducted under the operating conditions of 95% relative humidity, a feed temperature of 50 °C, a pressure difference of 10 kPa, a feed gas flux of 1 L/min, a sweeping gas flux of 2 L/min, and a sweeping gas temperature of 26 °C. Figure 4 shows that the greater effective area for the feed gas to contact the membrane leads to a higher water recovery rate in the membrane condenser. This is due to the increased contact area prolonging the duration of the feed gas interaction with the membrane module, allowing water molecules to fully contact the membrane surface and thereby improving the condensate transport flux and water recovery. When the membrane area is 43.71 cm^2^, the water recovery rate reaches 15.8%. As shown in Figure 4, the condensate transport flux remains relatively stable, with a decrease observed as the membrane area increases. While the condensate transport flux peaks at a membrane area of 10.93 cm^2^, the water recovery rate is lowest, indicating suboptimal conditions. Combining the water recovery rate and condensate transport flux, there is an optimal value for the membrane area of 32.78 cm^2^ and 43.71 cm^2^. The feed gas flux is then used to compare with the upper membrane area to estimate the required membrane area for actual conditions. The results indicate that the membrane condenser reaches the best effect in the ratio range from 0.51 to 0.67 cm/s.

### 3.3. Effect of Feed Gas Pressure

**Figure 5:**
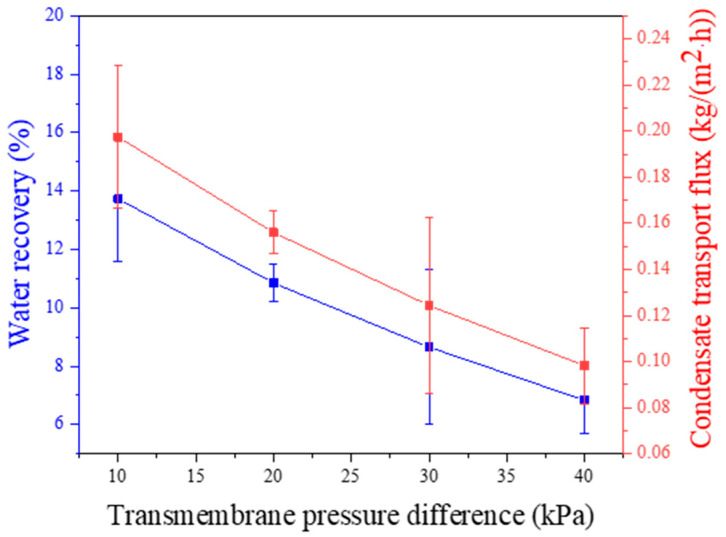
Effect of feed pressure on water recovery and condensate transport flux across membrane condenser (feed flow rate: 1 L/min).

This experiment was conducted under the operating conditions, including a relative humidity of 95%, a feed temperature of 50 °C, a feed gas flux of 1 L/min, a sweeping gas flux of 2 L/min, a sweeping gas temperature of 26 °C, and a membrane area of 32.78 cm^2^. As shown in Figure 5, it is observed that increasing the pressure on the feed side leads to a decrease in both the water recovery rate and condensing flow rate of the membrane condenser. This phenomenon occurs because higher operating pressure results in increased gas flow in contact with the membrane surface but also leads to an increase in feed gas passing through the membrane pores, carrying a significant amount of condensing water through the pores and resulting in a decrease in condensing flow. Therefore, the optimal feed pressure is determined to be 10 kPa, with a corresponding water recovery rate of 13.7%. 

### 3.4. Effect of Feed Gas Temperature

**Figure 6:**
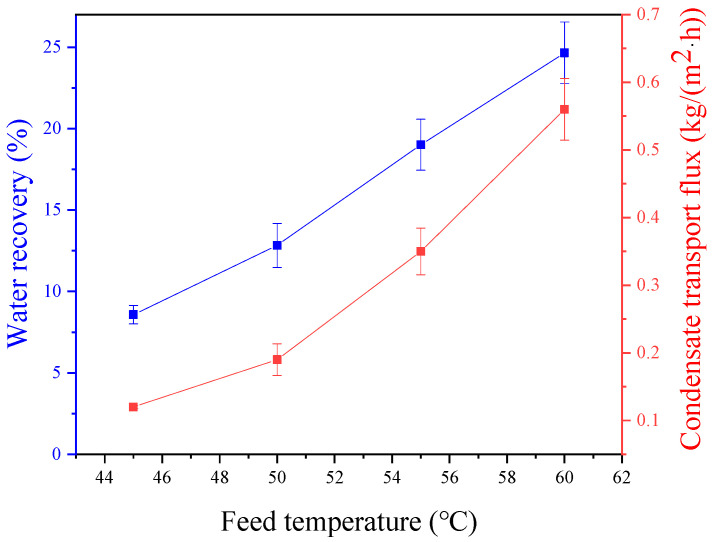
Effect of feed temperature on water recovery and condensate transport flux across membrane condenser.

This experiment was conducted under the operating conditions, including a relative humidity of 95%, a feed pressure of 10 kPa, a feed gas flux of 1 L/min, a sweeping gas flux of 2 L/min, a sweeping gas temperature of 26 °C, and a membrane area is 32.78 cm^2^. The recovery rate of membrane condensation increases as the feed temperature rises, as shown in Figure 6. The significant temperature difference between the two phases leads to a higher heat transfer driving force, facilitating heat exchange. With the same relative humidity, higher feed temperatures result in increased water vapor content, leading to condensation. At a feed temperature of 60 °C, the highest water recovery rate is 24.7%, but the increase trend is slightly reduced because the sweeping gas is constant, and the increasing temperature reduces the effect of cooling feed gas. Figure 6 shows the relationship between the feed temperature and the condensing flow rate. Higher feed temperatures correspond to higher gas moisture content and enthalpy values, increasing the molecular weight of condensed water per unit of time and enhancing heat transfer between the two phases. Additionally, the temperature difference rises, promoting mass and heat transfer in the membrane condenser process. Consequently, raising the feed temperature enhances the condensing flow. At a feed temperature of 60 °C, the flow rate of condensate water is 0.56 kg·m^−2^·h^−1^.

### 3.5. Effect of Sweeping Gas Flux

**Figure 7:**
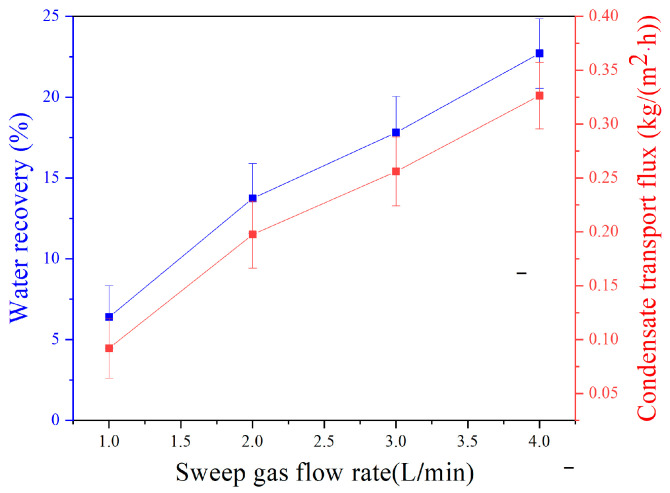
Effect of sweeping gas flow rate on water recovery and condensate transport flux across membrane condenser.

**Figure 8:**
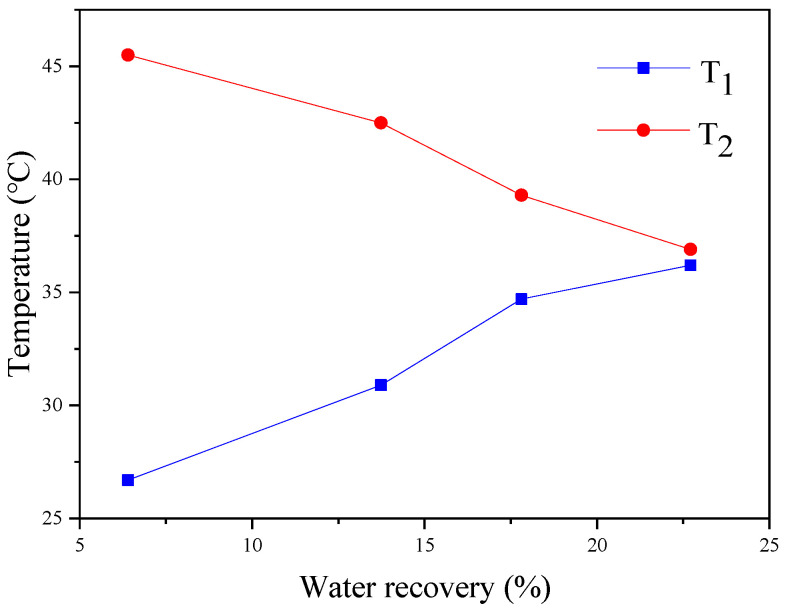
Effect of T_1_ and T_2_ on water recovery (T_1_: sweeping gas outlet temperature; T_2_: feed gas outlet temperature).

This experiment was conducted under the operating conditions with a relative humidity of 95%, a feed temperature of 50 °C, a feed pressure of 10 kPa, a feed gas flux of 1 L/min, a sweeping gas temperature of 26 °C, and a membrane area is 32.78 cm^2^. As shown in Figure 7, the increasing sweeping gas flux improves the water recovery rate and condensates transport flux. Specifically, at a sweeping gas flux of 4 L/min, the water recovery rate reached 22.7%, and the condensate flow was measured at 0.33 kg·m^−2^·h^−1^. The main reasons are divided into two aspects. On the one hand, increasing the sweeping gas flow rate can effectively enhance heat transfer by removing heat from the feed side, increasing the temperature difference between the two sides, and boosting the driving force for heat transfer. On the other hand, a high sweeping gas flow rate can reduce the thickness of the gas-phase boundary layer on the sweeping gas side and promote the process of heat transfer. The water recovery rate increases as the temperature difference between the sweeping gas side and the feed side decreases, while the inlet and outlet temperature differences decrease with an increase in sweeping gas flow. As shown in Figure 8, a higher sweeping gas flow rate can promote heat transfer and increase the condensing flow rate. However, it cannot ensure that the greater the sweeping gas flow rate is, the higher the condensate transport flux becomes. At a water recovery rate of 22.7%, a minimal temperature difference between the two sides indicates that the membrane condenser efficiency is near optimal levels. Further increasing the flow may not be advantageous and could lead to energy wastage. 

## 4. Conclusions

This study examines the effect of membrane condenser process operating parameters on water recovery and condensate transport flux. The operating parameters included feed gas temperature, feed gas flux, and inlet pressure. Sweeping gas flux and membrane area were altered for experimental analysis. The findings and discussions led to the following conclusions:

1. All investigated operational parameters have an impact on the membrane condenser process. Among them, the influence of feed gas temperature and sweeping gas flux is more significant compared to the effect of water recovery rate. At a temperature of 60 °C, the water recovery rate reaches 24.7%, while with a sweeping gas flux of 4 L/min, the water recovery rate reaches 22.7%;
2. When the intake flow rate is 1 L/min and 0.5 L/min, the water recovery rate exhibits an inverse relationship with the feed pressure. However, taking into account both the water recovery rate and condensing flow, the process parameters with a feed gas flux of 1 L/min and the feed pressure of 10 kPa are deemed optimal;
3. When the membrane area is held constant, the efficiency of the membrane condenser decreases with an increase in feed gas flux. Conversely, when the feed gas flux is constant, the efficiency of the membrane condenser increases with an increase in membrane area. This indicates a direct relationship between feed gas flux and membrane area. Experimental results suggest that the optimal relationship between the feed gas flux and membrane area falls within the range of 0.51–0.67 cm/s.

## Figures and Tables

**Table 1:** Basic parameters of membranes.

Membrane	Average Pore Size (nm)	Porosity (%)	Contact Angle (°)	Outer Diameter (mm)
PTFE	227.4 ± 7.1	42.12 ± 0.31	122.3 ± 1.6	1.74 ± 0.02

## Data Availability

The original contributions presented in the study are included in the article, further inquiries can be directed to the corresponding authors.
